# Supplementary material for: Effect of cellular rearrangement time delays on the rheology of vertex models for confluent tissues
Source: PLoS Comput Biol. 2021 Jun 7;17(6):e1009049. doi: 10.1371/journal.pcbi.1009049 (PMC8211246; doi:10.1371/journal.pcbi.1009049)
Supplement: S1 File — (PDF) [file pcbi.1009049.s001.pdf]

SUPPORTING INFORMATION

Effect of cellular rearrangement time delays on the rheology of vertex models for confluent tissues

Gonca Erdemci-Tandogan[1,2] and M. Lisa Manning[1,3]

[1]*Department of Physics, Syracuse University, Syracuse, New York, USA*
[2]*Institute of Biomedical Engineering, University of Toronto, Toronto, Ontario, Canada*
[3]*BioInspired Institute, Syracuse University, Syracuse, New York, USA*

The neighbors-overlap function

We define a neighbors-overlap function Q_n, as described in the text, which represents the fraction of cells that have lost two or more neighbors in time t. We decided upon the cutoff value of two or more neighbors based on the following observation. We compare the results obtained from the neighbors-overlap function with the results obtained from the self-overlap function. The two should give similar results for an isotropic tissue. The characteristic relaxation time obtained using the standard self-overlap function is similar to the one obtained from the neighbors-overlap function (Fig. S8(A)). On the contrary, a definition based on a cutoff value of losing three or more neighbors show a difference between the results obtained from the self-overlap function and that of the neighbors-overlap function (Fig. S8(B)).

Irreversible T1 transitions

In our model, cells go through a T1 transition whenever the edge l between four neighboring cells becomes less than a critical length l_c, and the T1 delay count is reached. The same group of four cells can go back and forth between their original configuration and their after T1 configuration until the final steady state condition is obtained (Fig. S7(A)). Hence, these flipping events can cause overcounting of the number of true T1 transitions. To avoid this, we calculate the number of successful T1 events where cells rearrange and stay in their new configurations. In other words, we calculate the number of irreversible T1 events.

To calculate the irreversible events (N_{irr}), we first characterize the reversible events (N_{rev}) in our simulations. When four cells go through a T1 transition, the two cells share an edge lose a vertex and the two cells that are not neighbor before the T1 transition gain a vertex (Fig. S7(A)). We record the cell configurations that go through the T1 transition and scan through the rest of the simulation steps to check whether the cell configurations are ever back to their "before T1" configurations in the next $\tau + t_{T1}$ steps. Here, τ is the natural time unit of the simulations. If the configuration is repeated, we count the event as reversible, and we call the time between the two same configurations reversibility time, t_R. As we are interested in instantaneous successful T1 events over time, we search for the reversibility in short timescales, namely in τ steps. Fig. S7(B) is a histogram of reversibility time for $t_{T1} = 0$ which fits to a power law (Fig. S7(B) inset) with a long tail which indicates that most of the flipping T1 events happen in very short timescales.

We calculate the fraction of irreversible events, $f = N_{irr}/N_{total}$. For an anisotropic tissue, 70% of the T1 events are irreversible for $t_{T1} = 0$ while almost all of the events are irreversible for $t_{T1} > 0$ (Fig. S7(C)). For an isotropic tissue, only 20% of the T1 transitions are irreversible for $t_{T1} = 0$ (Fig. S7(D)). The irreversibility increases as the t_{T1} delay time increases (Fig. S7(D)). To calculate the successful T1 events (main text Fig. 5(A) and (B)), we multiply the total number of T1 transitions by the fraction of irreversible events f (Fig. S7(C)) at each t_{T1} value. Similarly, for an isotropic tissue (Fig. S7(E)), we multiply the total number of T1 transitions by the fraction of irreversible events f (Fig. S7(D)) at each t_{T1} value.

Plateau value for the tissue aspect ratios in the absence of T1 transitions

As discussed in the main text and seen in Fig. 2(D), in the absence of T1 transitions (*i.e.*, at simulation timepoints before the T1 delay timescale) tissues under anisotropic tension will increase their aspect ratio up to a plateau value

and get stuck there. Numerical simulations confirm that this plateau value varies with both the isotropic tension $\kappa_P P_0$ in the bare vertex model and the additional anisotropic line tension γ_0 (Fig. S9).

To predict this plateau value analytically, we hypothesize that the plateau occurs when the shapes are at an energy minimum under the constraint there are no T1 transitions. We propose that the cells undergo a two-step process to minimize their energy, which is easiest to see starting from an ordered isotropic hexagonal packing, shown in Fig. S10(A). First, the large line tension on the vertical edges will cause those to shrink to zero length, generating a diamond pattern with 4-fold coordinated vertices, as shown in Fig. S10(B). This process should be independent of γ_0 and P_0 for values of γ_0 that are sufficiently large, and one can show it results in a change of aspect ratio by a factor of 1.58: $AR_1 = 1.58$.

Next, because that state is perfectly symmetric, the system can undergo a symmetry breaking so that some edges become shorter and closer to vertically-oriented, while alternating edges become longer and more horizontally oriented. In an ordered systems this generates some flag-shaped parallelograms, but in a disordered system one expects shapes that are roughly rectangular with the short sides oriented along the axis of higher tension, as shown in Fig. S10(C). To calculate the minimum energy state of such rectangles, we label the vertical length l_1 and horizontal length l_2, and assume the area of each cell is fixed to unity so that $l_2 = 1/l_1$. Taking the derivative of the part of the vertex energy functional related to line tensions, $E \sim \gamma_0 l_1 + \kappa_P (P - P_0)^2$, and then setting the derivative with respect to l_1 equal to zero results in a 4th order polynomial equation:

$$l_1^4 + \left(\frac{\gamma_0}{8\kappa_P} - \frac{P_0}{2}\right) l_1^3 + \frac{P_0}{2} l_1 - 1 = 0. \tag{1}$$

For a given values of κ_P, P_0, and γ, we can solve this equation for its positive roots and identify the energy minimizing value of l_1^{min}. The addition change to the global aspect ratio of the tissue allowed by these rectangles is simply ratio of $l_2 = 1/l_1$ to l_1: $AR_2 = 1/(l_1^{min})^2$.

Finally, the total change to the aspect ratio under both processes is simply $AR_{tot} = AR_1 \times AR_2 = 1.58/(l_1^{min})^2$. This analytic prediction is illustrated by the blue squares in Fig. S10(D). The observed plateau values are given by the red circles, and they are in fairly good agreement. The analytic prediction overestimates the aspect ratio for the lowest value of γ_0, likely because the tension isn't sufficiently large to shrink vertical edges to zero in the first process.

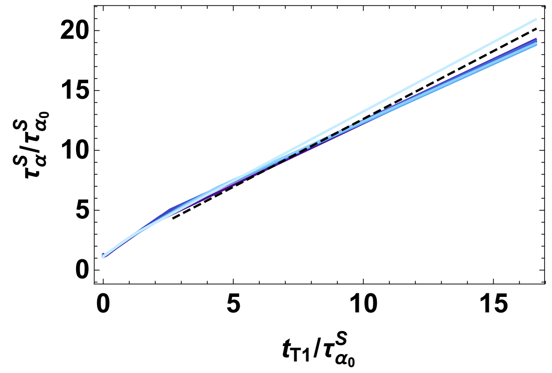

FIG. S1. The characteristic relaxation time on a linear scale. The characteristic relaxation time τ_α^S on linear scale (log-log scale as in Fig. 1) as a function of T1 delay time normalized by the collective response timescale $\tau_{\alpha 0}^S$ without T1 delay. The dashed line is the best linear fit to high T1 delay region with a slope of $m = 1.13$. Colors correspond to different values of $p_0 = 3.74, 3.76, 3.78...3.9$ (darker to light blue), for fixed $T = 0.02$, and $N = 256$.

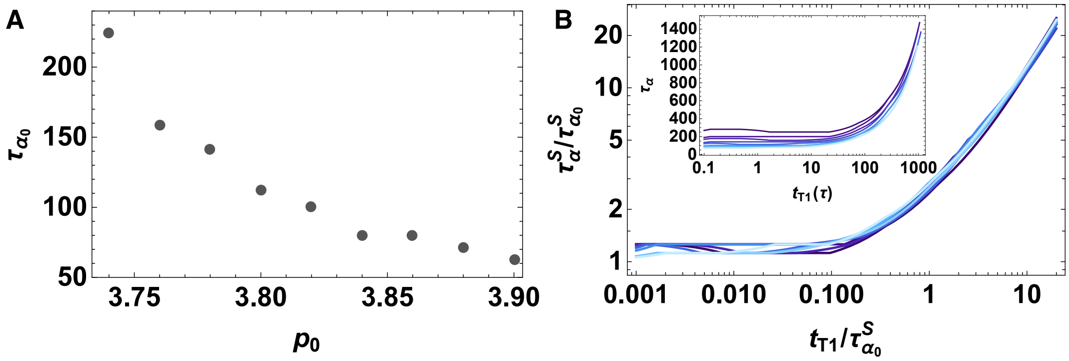

FIG. S2. The characteristic relaxation time depends on the cell shape. A) The characteristic relaxation time in the absence of T1 delays, defined by the self-overlap function, for various p_0 values for a system size with $N = 1024$. The tissue becomes more viscous as p0 decreases at fixed temperature $T = 0.02$. B) Log-log plot showing collapse of the characteristic relaxation time τ_α^S as a function of T1 delay time normalized by the collective response timescale $\tau_{\alpha 0}^S$ without a T1 delay. Colors correspond to different values of $p_0 = 3.74, 3.76, 3.78...3.9$ (darker to light blue), for fixed $T = 0.02$, and $N = 1024$. The inset shows the characteristic relaxation time τ_α^S as a function of T1 delay time without any normalization, for the same values of p_0..

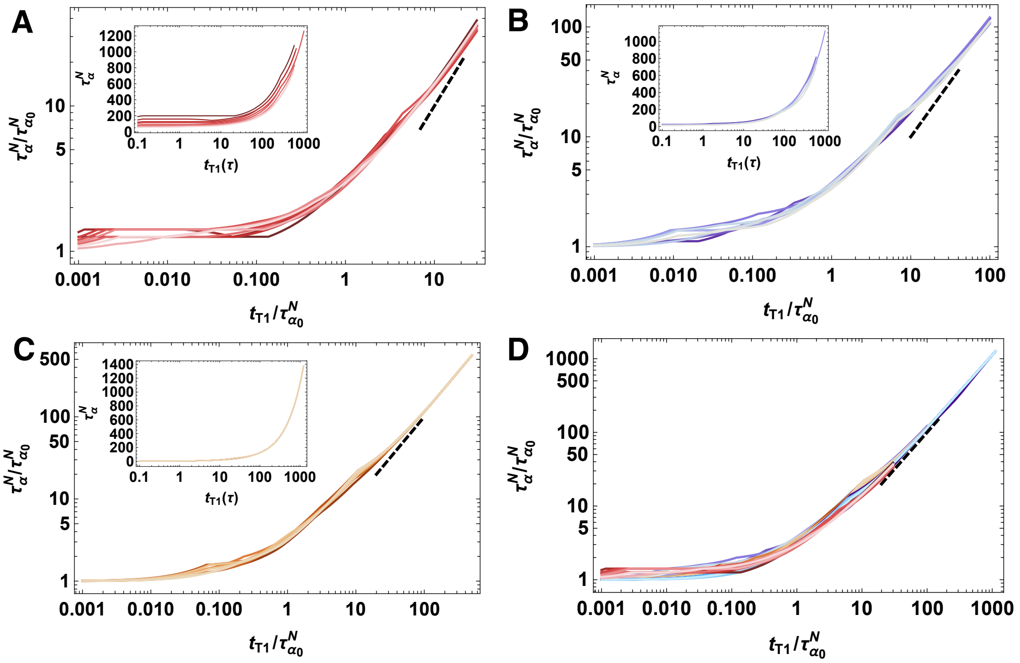

FIG. S3. The behavior of the characteristic relaxation time is independent of the magnitude of the line tension in the anisotropic model. A) The characteristic relaxation time τ_α^N as a function of T1 rearrangement delay time for anisotropic tissue simulations with anisotropic line tension amplitude of $\gamma_0 = 0.01$ (A), $\gamma_0 = 0.1$ (B) and $\gamma_0 = 0.5$ (C). D) Overlap of the data in (A), (B), (C) and Fig. 2(C). Darker to lighter tones for each color represents the data for $p_0 = 3.74, 3.76, 3.78...3.9$, and other parameters are $T = 0.02$, and $N = 256$. Each data set are normalized by the corresponding characteristic relaxation time $\tau_{\alpha 0}^N$ where the T1 rearrangement is instantaneous, $t_{T1} = 0$.

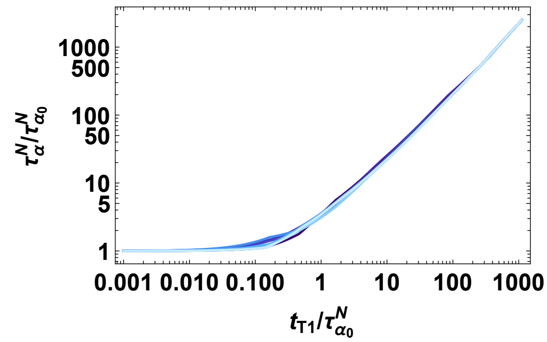

FIG. S4. The behavior of the characteristic relaxation time is the same at zero temperature. Data collapse for $p_0 = 3.74.376, 3.78...3.9$ (darker to lighter blue), $N = 256$ and $\gamma_0 = 1.0$ at zero temperature. The characteristic relaxation time, τ_α^N as a function of T1 rearrangement delay time normalized by the collective response timescale $\tau_{\alpha 0}^N(t_{T1} = 0)$.

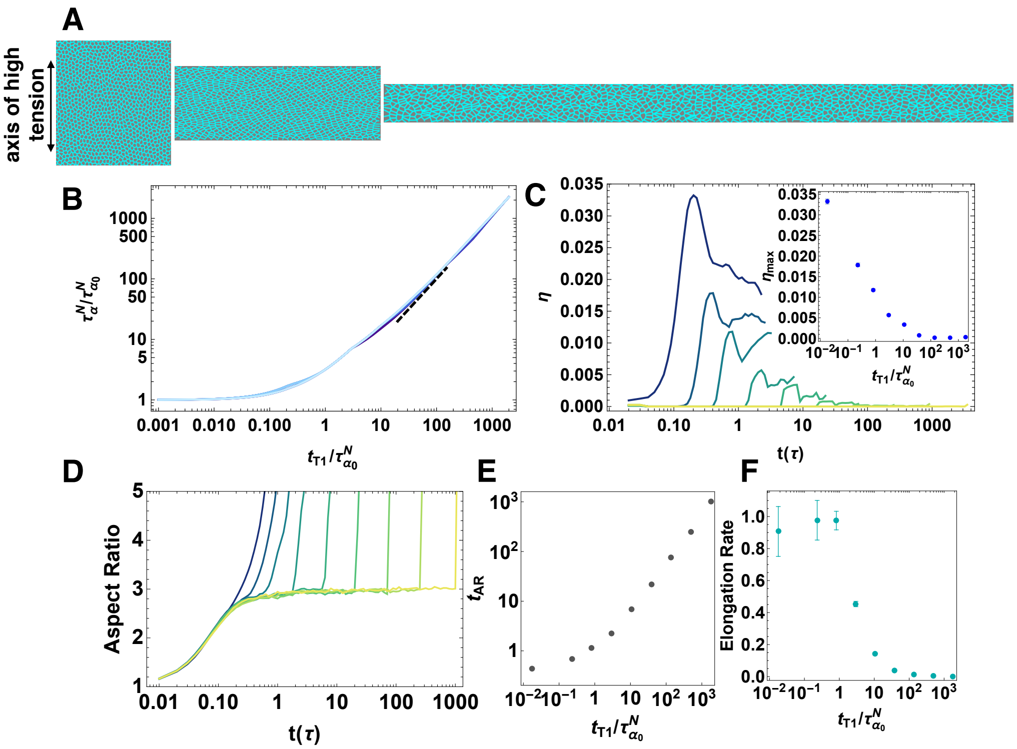

FIG. S5. Simulations of an anisotropic tissue with a larger system size. A) An anisotropic line tension on vertical edges is introduced to obtain global anisotropic changes to tissue shape. B) Data collapse for $p_0 = 3.74 3.76, 3.78...3.9$ (darker to light blue), $T = 0.02$, $N = 1024$ and $\gamma_0 = 1.0$. The characteristic relaxation time, τ_α^N as a function of T1 rearrangement delay time normalized by the collective response timescale $\tau_{\alpha 0}(t_{T1} = 0)$. The dotted line is a slope of 1. C) The number of T1 transitions per cell over time and at the maximum averaged over 10 realizations (inset) for T1 delay time of $t_{T1} = 0$, 0.13, 0.46, 1.67, 5.99, 21.5, 77.4, 278.2 and 1000 τ (dark green to yellow), $p_0 = 3.74$, $T = 0.02$, $N = 1024$ and $\gamma_0 = 1.0$. D) The aspect ratio of the simulation box over time for T1 delay time of $t_{T1} = 0$, 0.13, 0.46, 1.67, 5.99, 21.5, 77.4, 278.2 and 1000 τ (dark green to yellow), $p_0 = 3.74$, $T = 0.02$, $N = 1024$ and $\gamma_0 = 1.0$. E) The time (t_{AR}) at which the system first goes above the plateau value as a function of t_{T1} for each aspect ratio curve in (D). F) The rate of elongation obtained from the aspect ratio curves in (D) as a function of t_{T1} delay time. Both (C), (D), (E) and (F) are from 10 realizations. Error bars represent one standard error.

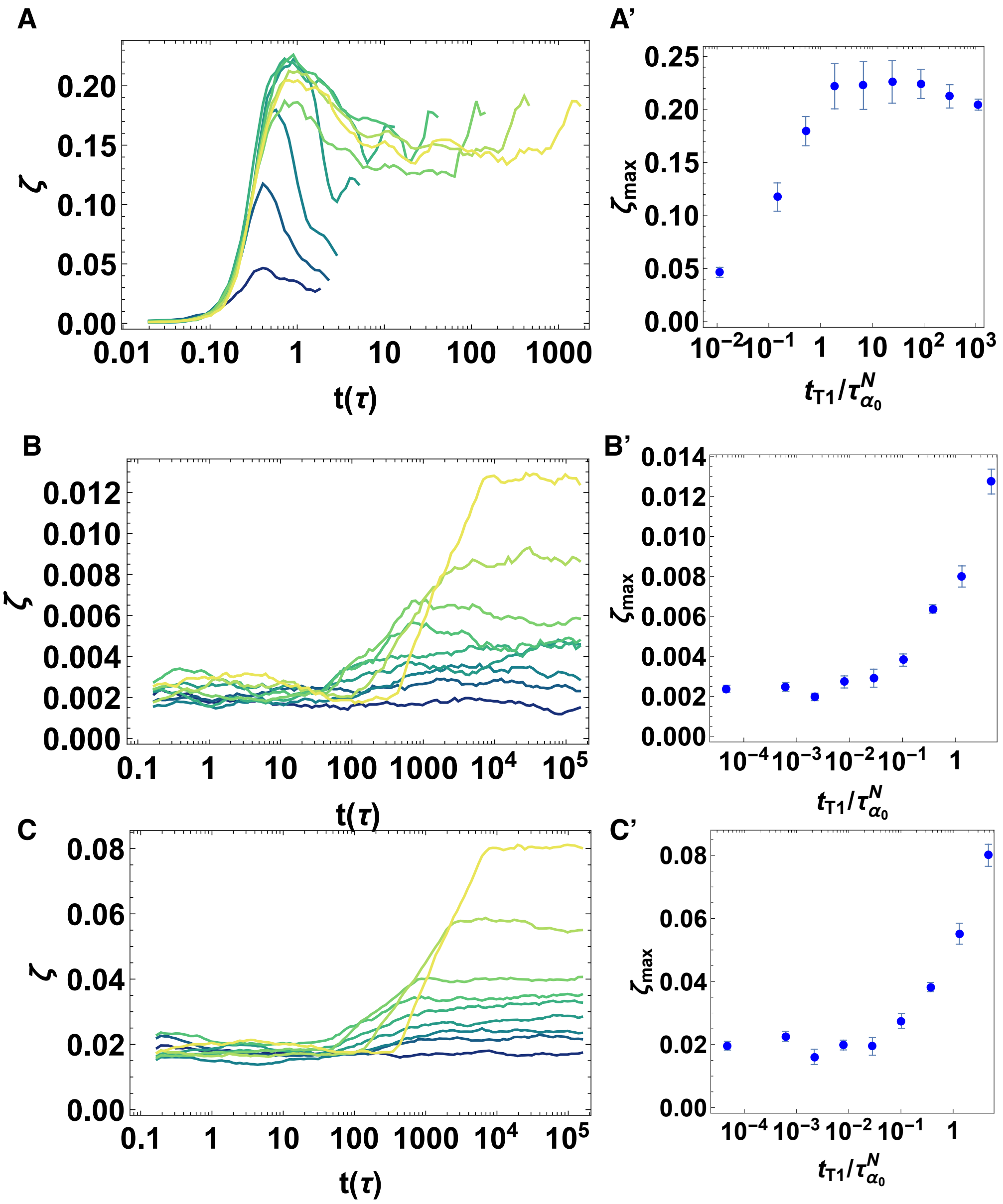

FIG. S6. Counting very short edges as a proxy for many-fold vertices. Number of very short edges per cell ξ for an anisotropic tissue as a function of time (A). Shaded lines represent different T1 delay times $t_{T1} = 0$, 0.13, 0.46, 1.67, 5.99, 21.5, 77.4, 278.2 and 1000 τ (dark green to yellow), for a tissue with $\tau^N_{\alpha 0} = 0.89\tau - p_0 = 3.74$, $T = 0.02$, $N = 256$ and $\gamma_0 = 1.0$. The cutoff value to threshold very short edges as a proxy for multi-fold coordination is $0.04\sqrt{A_0}$. (A') Ensemble-averaged maximum value of ξ over a simulation timecourse vs. the T1 delay time t_{T1} normalized by $\tau^N_{\alpha 0}$. The average is taken over 10 independent simulations, and error bars correspond to one standard error. Number of very short edges per cell ξ over time (B, C) and the ensemble-averaged maximum value of ξ over a simulation timecourse (B', C') for an isotropic tissue simulations of T1 delay time of $t_{T1} = 0$, 0.13, 0.46, 1.67, 5.99, 21.5, 77.4, 278.2 and 1000 τ (dark green to yellow), $p_0 = 3.74$, $T = 0.02$ and $N = 256$. The cutoff value to threshold very short edges as a proxy for multi-fold coordination is $0.04\sqrt{A_0}$ in (B, B') and $0.11\sqrt{A_0}$ in (C, C'). The average is taken over 10 independent simulations, and error bars correspond to one standard error.

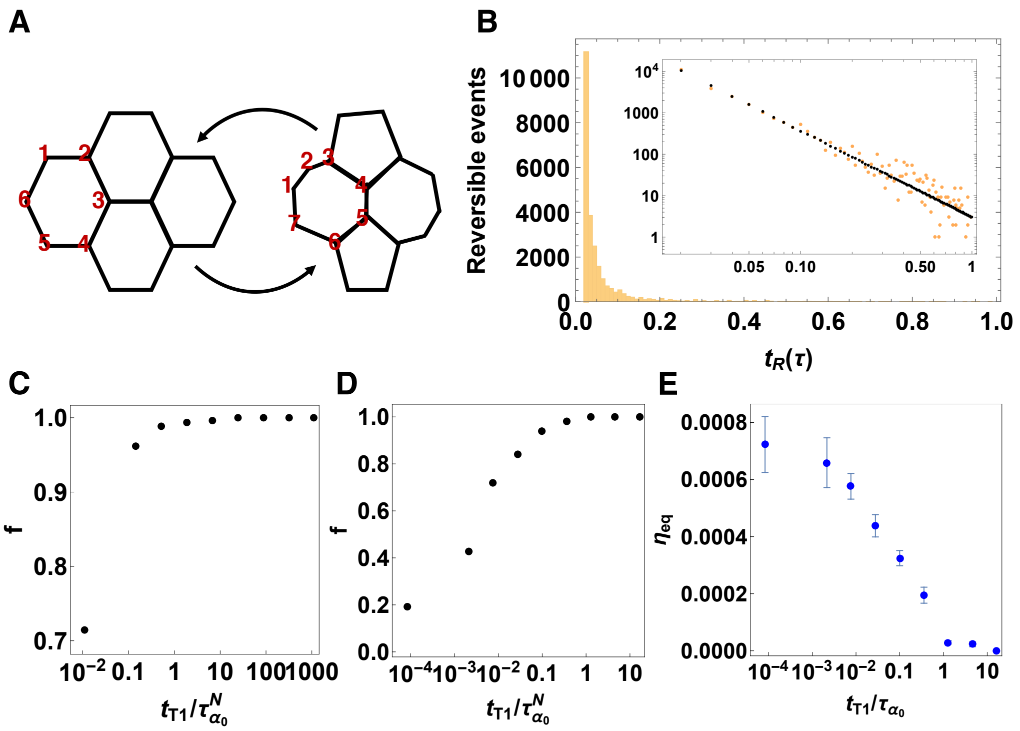

FIG. S7. Number of successful T1 transitions. A) Schematic of a reversible T1 transition. The two cells that are neighbor before the T1 transition lose a vertex (top and bottom cells) and the cells that are not neighbors before the T1 transition gain a vertex and become neighbors (right and left cells). Vertices of the cell on the left are labeled as 1,2,...6. The same cell has a configuration of vertices as 1,2,...7 after the T1 transition. If the T1 transition is reversible, then the cell goes back to its original configuration of vertices 1,2,...6. We track such cell configuration changes in our simulations to determine if a T1 event is reversed. B) Histogram of reversibility time t_R which fits to a power law (inset black points) for an anisotropic tissue of $p_0 = 3.74$, $T = 0.02$, $N = 256$, $t_{T1} = 0$ and $\gamma_0 = 1.0$. C) Fraction of irreversible events as a function of T1 delay time for an anisotropic tissue of $p_0 = 3.74$, $T = 0.02$, $N = 256$ and $\gamma_0 = 1.0$. D) Fraction of irreversible events as a function of T1 delay time for an isotropic tissue of $p_0 = 3.9$, $T = 0.02$ and $N = 256$. E) Number of successful (irreversible) T1 transitions at the equilibrium averaged over 10 realizations for an isotropic tissue of $p_0 = 3.9$, $T = 0.02$ and $N = 256$. Error bars represent one standard error.

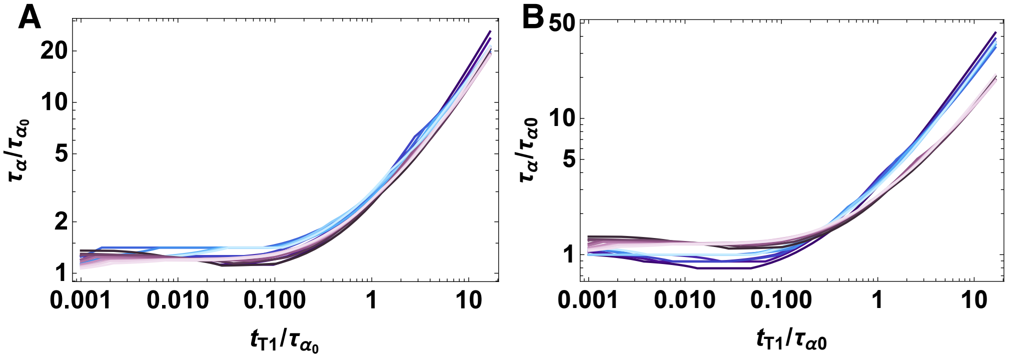

FIG. S8. The characteristic relaxation time obtained using the standard self-overlap function vs. the neighbors-overlap function. The characteristic relaxation time, τ_α, obtained using neighbors-overlap function (based on losing 2 or more neighbors (A) and losing 3 or more neighbors (B)), as a function of T1 rearrangement time for $p_0 = 3.74, 3.76, 3.78...3.9$ (darker to light blue) and $T = 0.02$ for an isotropic tissue. Both (A) and (B) are plotted together with the characteristic relaxation time obtained using the self-overlap function for comparison for $p_0 = 3.74, 3.76, 3.78...3.9$ (dark purple to light pink). Each data set are normalized by the corresponding characteristic relaxation time $\tau_{\alpha 0}$ where the T1 rearrangement is instantaneous, $t_{T1} = 0$.

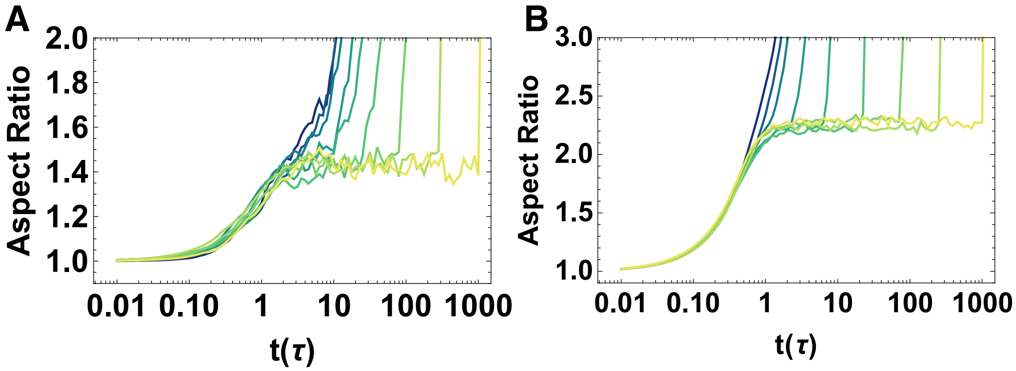

FIG. S9. Aspect ratio plateau value varies with the additional anisotropic line tension γ_0. The aspect ratio of the simulation box over time for T1 delay time of t_{T1} = 0, 0.13, 0.46, 1.67, 5.99, 21.5, 77.4, 278.2 and 1000 τ (dark green to yellow), $p_0 = 3.74$, $T = 0.02$ and $N = 256$. The anisotropic line tension amplitude $\gamma_0 = 0.1$ (A) and $\gamma_0 = 0.5$ (B). The data in (A) plateau at aspect ratio of ~ 1.4 and the data in (B) plateau at aspect ratio of ~ 2.2.

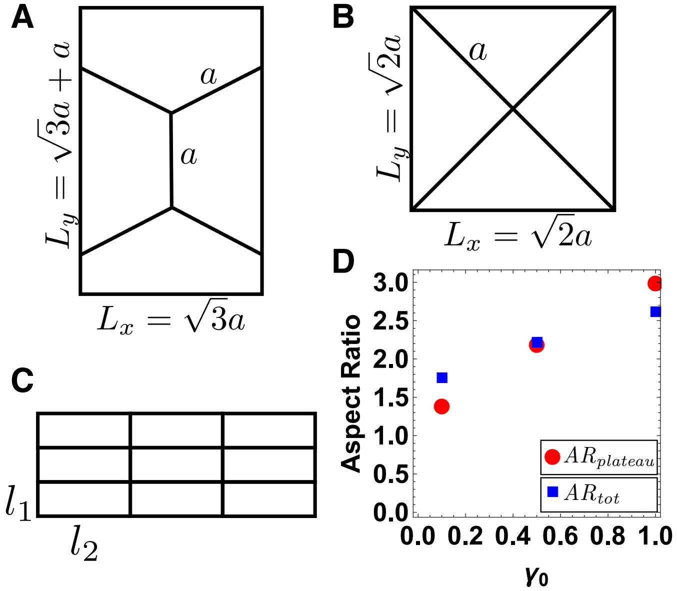

FIG. S10. Analytical prediction for the plateau values of the aspect ratio as a function of time. A) A hexagonal unit box with a hexagon side of a. B) The cells meet at a 4-fold coordinated vertex after a high tension is applied on the vertical edges of the hexagons in (A). Cells form a diamond pattern with a side length of a. Both in (A) and (B), L_x and L_y are the sides of the box along the horizontal and vertical direction. C) A rectangular grid of cells with sides l_1 along the vertical and l_2 along the horizontal direction. D) Analytical prediction of the plateau values observed in simulations with various γ_0 values (Fig. 2D, Fig. S9A, Fig. S9B). Red circles correspond to the plateau values in the simulations. Blue squares correspond to the analytical prediction for the total change to the aspect ratio as described in Supporting Information text.
