# Supplementary material for: Effect of cellular rearrangement time delays on the rheology of vertex models for confluent tissues
Source: PLoS Comput Biol. 2021 Jun 7;17(6):e1009049. doi: 10.1371/journal.pcbi.1009049 (PMC8211246; doi:10.1371/journal.pcbi.1009049)
Supplement: Attachment — Submitted filename: CoverLetter_and_Response_to_Reviewers_Updated.pdf [file pcbi.1009049.s002.pdf]

April 15, 2021

Dear Dr. Nie,

Thank you very much for sending us the reviewer reports on our manuscript PCOMPBIOL-D-21-00286, entitled "Effect of cellular rearrangement time delays on the rheology of vertex models for confluent tissues". We are very pleased that both reviewers find our manuscript of high interest to the field and recommend the paper for publication.

We thank the reviewers for their careful consideration of our manuscript, and their helpful suggestions. We have included a point-by-point response below and have revised the manuscript based on the comments and questions raised by reviewers. Since we have addressed all the points of the reviewers in the manuscript, we hope that the editor finds the revised manuscript suitable for publication.

We would like to note that we chose to call the Methods section Model and include it before the Results section as the guidelines allow those choices and it is important to define the parameters and model before the Results section to make our paper clearer to the readers.

We look forward to hearing from you.

Sincerely,

Gonca Erdemci-Tandogan
Lisa Manning

<u>Response to Reviewers:</u>

Reviewer #1:

This manuscript reports an implementation to the classical vertex model that will have an important impact for simulating remodelling of epithelial tissue morphogenesis. The authors include indeed a time delay that corresponds to the most sensitive (and still quite mysterious) step in cell intercalation, i.e. the famous T1 transition. This step corresponds to the phase where the shrinking contacts has brought the two vertices so close that they seemingly fuse into a single four-fold vertex that may either regress (aborted transition), or successfully resolve in neighbour exchange with formation of a new contact. So far, vertex models have considered this step as instantaneous. Yes, at the cellular level, this transition is certainly not a straightforward mechanism. One may rather expect it to be complex, time- and energy-consuming, thus. The authors go on to explore the consequences of including this delay, and discover a very simple relationship between the length of this delay and the time scale of global tissue response. One very interesting consequence suggested by the authors is that by mechanisms that may slow down T1 transitions are predicted to stiffen the tissue. I would add that conversely, the still

unknown molecular events required to remodel and/or dismount two tricellular junctions of an epithelium to build a new contact are bond to constitute a general rate limiting factor to epithelial morphogenesis. While I can commend the high interest of this manuscript in the field of morphogenesis, I should specify that I am not a physicist, thus I am not able to critically evaluate the detailed mathematical aspects of the manuscript, although I do understand the general features of the model, the basic formulae and the implemented parameters, which I find fully adequate.

We thank the reviewer for their comments regarding the high interest of this work to the field and their overall positive view of our work.

Reviewer #2: In their manuscript, Erdemci-Tandogan and Manning studied how cellular rearrangement delays might affect the dynamics of confluent tissues. In confluent tissues, where cells occupy the entire space, the neighbor exchange is the dominant mode for large-scale movement of cells in the tissue. In 2D, this process is modeled by so-called T1 transition, where the boundary between two cells shrinks to zero, the local connectivity of the four cells surrounding this boundary changes, which then follows by an expansion of the new boundary. In original Vertex models, this process is taken ad hoc, with no details that might reflect the rearrangement of proteins in a cell boundary going through a T1 transition. Such considerations are necessary to more precisely understand tissue dynamics. In their manuscript, Erdemci-Tandogan and Manning present a modification of the T1 transition, where the rearrangement is delayed. Such delay has several consequences on tissue remodeling, including the accumulation of four-way and higher coordination vertices, and more generally, the tissue dynamics. I found the results in this manuscript interesting and worth publishing in PLOS Computational Biology. However, I have a few suggestions and comments that might help the readers:

We thank the reviewer for highlighting our results as interesting.

1- The authors used a time delay in T1 transitions by explicitly preventing the shrinking boundaries from going through rearrangement. Another way to include such delay would be to assign an energy barrier for the T1 transitions. This has the advantage of a slight modification to the original Vertex model energy function but keeping the dynamics unchanged. Could the authors elaborate if an explicit delay in T1 transitions is a more appropriate way of modeling the biological details of cellular rearrangements than other possible models such as an energy barrier?

This is an interesting suggestion. We considered something similar initially, but discarded it in favor of a T1 delay timescale for a few reasons.

First, the potential energy landscape of the vertex model is extremely high-dimensional (ostensibly the number of vertices times two – the number of spatial dimensions). We often (as in Bi et al Nat Phys 2015) represent the energy barrier along a reaction coordinate corresponding to the distance between the two vertices between the edge that is shrinking during the T1, though that is a simplification. We have previously established that there is a saddle point precisely when that edge gets to zero length.

So, we could add a constant energy barrier either (i) as a delta function with a specified total area precisely at that saddle point, or (ii) we could model it as a gaussian or other function with compact support in that high-dimensional space, centered at the saddle. For choice (i), with finite temperature dynamics of the sort we simulate here, then after the edge shrinks to zero length we would expect T1 transitions according to an Arrhenius rate which is exponential in the height of the energy barrier/ kb T, and gives rise to a characteristic timescale governed by the magnitude of the energy barrier. This energy barrier approach would be very similar to what we already simulated in the manuscript (i.e. a fixed characteristic delay timescale), but it may introduce some noise into the system because the actual delay time would be stochastic around the average. In other words, the delta function energy barrier would "turn on" right at the point where the edge shrank to zero, just like our time delay "turns on" when an edge shrinks to zero length.

If instead we opted for choice (ii), there are multiple parameters that we would have to specify – is the additional energy barrier symmetric in all directions? Much larger or smaller in magnitude along the reaction coordinate? What is the length scale associated with the spread of the function in coordinate space? We are not sure how to parameterize these functions or how to link them back to experimental observables.

Another possibility is to not consider a constant energy barrier, but instead one that depends on the edge length itself (for example, adding a spring-like term that resists compression of a shrinking edge, or a mechano-sensitive edge that will only shrink when the tension gets high enough). The latter mechanism has already been studied by Banerjee and collaborators, and we reference it in our manuscript (old ref 28). The behavior of such models is clearly different from the class of models we study here, as it would generate a strong feedback between cell shape and the effective T1 delay time (since cell shape alters local tension in the network). A virtue of our model is that it tests the limit where the cell-shape-driven tissue fluidity and the localized mechanisms that delay the rearrangements are explicitly independent, allowing us to study the effect of each.

For all of these reasons, we have chosen to focus on the T1 delay time instead of an energy-barrier-based perturbation. We have added a sentence to the main text stating that we focus on T1 delay time instead of an altered energy barrier between mechanically stable states for simplicity.

2- I understand the origin of thermal noise in the context of molecular dynamic simulations of passive particles. However, I do not understand the origin of thermal fluctuations of vertices in the context of confluent tissues. Is this just a modeling concept, or is it referring to a biological process? Also, do the results fundamentally different at zero temperature? If not, why the authors did not consider studying their model in zero temperature, which is one less parameter to worry about.

The "finite temperature" fluctuations we study here are meant to capture a specific set of biological processes, namely the fluctuations of vertex positions generated by active processes in the cell (i.e. these fluctuations drop significantly in the absence of ATP). Therefore, the temperature we specify is not the real thermal temperature measured by a thermometer, but

instead an effective temperature meant to capture important features of these active driving forces.

Although the origin of vertex fluctuations in dense tissues is an active area of study, actomyosin cytoskeletal components, and E-cad levels fluctuate as the cell edge lengths fluctuate [Fodor et al. Biophysical Journal 114, 939–946 (2018), Rauzi et al. Nature 468, 1110-1114 (2010), Fernandez-Gonzalez et al. Developmental Cell 17, 736–743 (2009)]. A recent study also emphasizes that the deviation of neighboring cells in the early embryogenesis of the zebrafish resembles Brownian motion supporting the common usage of white noise fluctuations in modeling of cells [Simoes et al. arXiv:1911.04782 (2019)]. In addition, the convergent extension movement we model in this paper exhibits fluctuations on cell vertices [Fernandez-Gonzalez et al. Developmental Cell17, 736–743 (2009)]. Therefore, we focus on the simulations with finite temperature.

In isotropic systems, such fluctuations are necessary to explore the configuration space and allow the tissue to flow. However, in systems where there are anisotropic forces, those anisotropic forces can cause the tissue to flow and so we can study the system at zero temperature. Therefore, in response to this referee request, we have now extensively simulated the anisotropic vertex model in the limit of zero temperature. The results are shown in Figure 1 below, and are very similar to the finite temperature simulations for anisotropic systems shown in the manuscript. We have added a sentence to the main text highlighting that anisotropic systems exhibit the same behavior at zero temperature, and we also added this figure to the supplemental material.

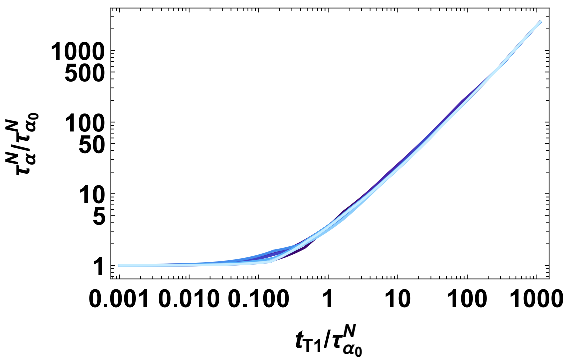

Figure 1. Simulations of an anisotropic tissue at zero temperature. The characteristic relaxation time as a function of T1 rearrangement delay time for p0 = 3.74, 3.76, 3.78...3.9 (dark blue to light blue), T = 0, N=256 and gamma0=1.0.

3- In modeling anisotropic tension and tissue shear, the authors considered a fairly small box for such a large deformation. There might be finite-size effects due to periodic boundary conditions. I suggest the authors check this by repeating some of the simulations for different box sizes (i.e., number of cells) to ensure the results do not depend on box size.

We agree with the reviewer that finite size effects are an important consideration, and the reviewer is correct that a significant fraction of the data we show in the main text is for relatively

small system sizes with N=256. To study the impact of different system sizes (number of cells) on our results, we already simulated the anisotropic model with a larger number of cells -- N=1024 (old version Fig. S3, new version Fig. S5). The results with N=1024 were very similar to the results we obtained with N=256 (Figure 2), suggesting that finite-size effects are not dominating our results for anisotropic systems. Based on the reviewer's suggestion, we have now checked whether finite-size effects are important for the isotropic model, with exensive additional simulations. This new data, shown in the figure pasted below, demonstrates that the isotropic model exhibits the same behavior for the characteristic relaxation time with N=1024 cells as smaller systems with N=256 cells.

We have added a sentence to the main text that our results are not dependent on the system size, and we have added the figure for the N=1024 size isotropic systems to the supplemental material.

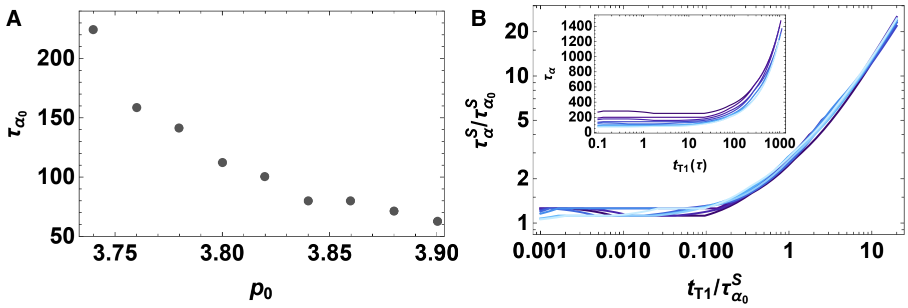

Figure 2. The characteristic relaxation time in the absence of T1 delays, defined by the self-overlap function, for various p0 values for a system size with $N=1024$. B) Log-log plot showing collapse of the characteristic relaxation time as a function of T1 delay time normalized by the collective response timescale without a T1 delay. Colors correspond to different values of p_0=3.74,3.76,3.78...3.9 (darker to light blue), for fixed $T=0.02$, and $N=1024$. The inset shows the characteristic relaxation time as a function of T1 delay time without any normalization, for the same values of p_0.

4- While the authors performed a detailed analysis of T1 transitions in their simulations, it would be interesting to connect those to more coarse-grained properties of the network, such as shear and bulk moduli. Is it possible to measure the shear modulus of the network as a function of T1 delay, and if so, I recommend incorporating it in the manuscript.

This is an astute question from the reviewer, and related to their question #1 which we answered in detail above. Specifically, as the reviewer correctly pointed out in that question, the T1 delay time alters the dynamics of the tissue, but not the mechanical energy. Therefore, linear response properties, such as the bulk and shear moduli, are not affected by the T1 delay time.

Specifically, based on the reviewer's suggestion, we actually did go back and calculate the shear modulus for all of the systems in our manuscript (we reported similar shear modulus measurements in the absence of T1 delays in the paper by Wang et al PNAS 2020 where we were co-authors). We generically find that in systems with T1 delays, the cellular networks display elongated cell shapes that would be associated with fluid-like behavior in the absence of

T1 delays. Therefore, when we calculate the shear modulus (the response to infinitesimal perturbations) we find that all of the systems formally have zero shear modulus.

A more interesting calculation would be to study the dynamic modulus as a function of an applied periodic perturbation with a specific frequency and amplitude, as is done in rheology studies of colloids. In that case, since we are explicitly studying the dynamical response to finite driving rates, this dynamic modulus would incorporate the effects of T1 delays.

Importantly, previous work on colloid rheology (Mason and Weitz, https://journals.aps.org/prl/abstract/10.1103/PhysRevLett.74.1250, which we now cite in the updated manuscript, new reference 43) has confirmed that the dynamic storage and loss moduli (the real and imaginary parts of the complex shear modulus) measured by oscillation experiments match up precisely with particle-motion observations-- such as the mean-squared displacement or the self-overlap function – which is what we study in this manuscript (e.g Fig 1D).

In addition, developing numerical code that applies wavelike perturbations of a given magnitude and frequency and quantifying the response is a highly non-trivial undertaking, and significantly beyond the scope of this work. However, it could be an interesting avenue for future research, and in fact we know some research groups (including the group of Frank Julicher, with a preprint we have now cited, new reference 42) are currently studying the nonlinear rheology of the bare vertex model in the absence of T1 delays, which is a task difficult enough to merit its own paper.

Taken together, this suggests that our analysis of the self-overlap function is likely sufficient to indicate the dynamic modulus of the tissue, but that more work (specifically, nonlinear rheology measurements of the vertex model with T1 time delays) should be performed in the future to confirm this. We have added a paragraph to the discussion to highlight this point.

5- In the simulations of anisotropic tension, the authors change the tension on a boundary based on its angle relative to an axis in the tissue. As the tissue evolves and the boundaries change orientation, their tension instantaneously changes. However, changes in the tension of a boundary require the actomyosin rearrangement, which also might happen with its timescales and delays

As we discussed in the Discussion section, and in our response to question #1 above, our work focuses on the effects of a fixed T1 delay time, but it would be interesting to study a T1 delay that is dependent on the local mechanical features of the cells. For example, updating the T1 delay time based on the tension on the edges (e.g. as a result of the actomyosin rearrangement as the reviewer suggested) would cause heterogeneity in the system. Such heterogeneity could lead to interesting tissue behavior; however, it is beyond the scope of this manuscript.
